# Incidence and pattern of urgent revascularization in acute coronary syndromes treated with ticagrelor or prasugrel

**DOI:** 10.1007/s00392-024-02454-x

**Published:** 2024-05-13

**Authors:** Alp Aytekin, Maria Scalamogna, J. J. Coughlan, Shqipdona Lahu, Gjin Ndrepepa, Maurizio Menichelli, Katharina Mayer, Jochen Wöhrle, Isabell Bernlochner, Bernhard Witzenbichler, Willibald Hochholzer, Dirk Sibbing, Dominick J. Angiolillo, Rayyan Hemetsberger, Ralph Tölg, Christian Valina, Arne Müller, Sebastian Kufner, Christoph Liebetrau, Erion Xhepa, Alexander Hapfelmeier, Hendrik B. Sager, Michael Joner, Gert Richardt, Karl-Ludwig Laugwitz, Franz Josef Neumann, Heribert Schunkert, Stefanie Schüpke, Adnan Kastrati, Salvatore Cassese

**Affiliations:** 1 Department of Cardiology, Deutsches Herzzentrum München, Technische Universität München, Lazarettstrasse, 36, Munich, Germany; 2 Cardiovascular Research Institute, Mater Private Network, Dublin, Ireland; 3 Department of Cardiology, Ospedale Fabrizio Spaziani, Frosinone, Italy; 4 Department of Cardiology, Medical Campus Lake Constance, Friedrichshafen, Germany; 5 Klinik Und Poliklinik Für Innere Medizin I, Klinikum Rechts Der Isar, Technische Universität München, Munich, Germany; 6 German Center for Cardiovascular Research, Partner Site Munich Heart Alliance, Munich, Germany; 7 Department of Cardiology and Pneumology, Helios Amper-Klinikum Dachau, Dachau, Germany; 8 Department of Cardiology and Intensive Care Medicine, Klinikum Würzburg Mitte, Würzburg, Germany; 9 Department of Cardiology, Klinik der Universität München, Ludwig–Maximilians–University, Munich, Germany; 10 Division of Cardiology, University of Florida College of Medicine, Jacksonville, FL USA; 11 Department of Cardiology, Internal Medicine II, Medical University of Vienna, Vienna, Austria; 12 Department of Cardiology, Heart Center Bad Segeberg, Bad Segeberg, Germany; 13 Department of Cardiology and Angiology, Medical Center - University of Freiburg, Freiburg, Germany; 14 Faculty of Medicine, University of Freiburg, Freiburg, Germany; 15 Heart Center, Campus Kerckhoff of Justus-Liebig-University, Giessen, Germany; 16 Institute of AI and Informatics in Medicine, School of Medicine, Technical University of Munich, Munich, Germany; 17 School of Medicine, Institute of General Practice and Health Services Research, Munich, Germany

**Keywords:** Antiplatelet therapy, Drug-eluting stent, Urgent revascularization, Percutaneous coronary intervention

## Abstract

**Background:**

The ISAR-REACT 5 trial compared the efficacy and safety of ticagrelor and prasugrel in patients with ACS managed invasively. The present study sought to investigate the impact of ticagrelor and prasugrel on the incidence and pattern of urgent revascularization in acute coronary syndromes (ACS) patients undergoing percutaneous coronary intervention (PCI).

**Methods and results:**

This post-hoc analysis of the ISAR-REACT 5 trial included all ACS patients who underwent PCI. The primary endpoint for this analysis was the incidence of urgent revascularization at 12-month follow-up. Secondary outcome was the pattern of urgent revascularization procedures (namely, urgent target vessel/non-target vessel revascularization – TVR/NTVR). Among 3,377 ACS patients who underwent PCI, 1,676 were assigned to ticagrelor and 1,701 to prasugrel before PCI. After 12 months, the incidence of urgent revascularization was higher among patients assigned to ticagrelor as compared to prasugrel (6.8% vs. 5.2%; hazard ratio [HR] = 1.32, 95% confidence interval [CI] 1.00–1.75; *p* = 0.051), mostly attributable to significantly more urgent NTVR in the ticagrelor group (3.8% vs. 2.4%; HR = 1.62 [1.09–2.41]; *p* = 0.017). The risk of urgent TVR did not differ between treatment groups (3.3% vs. 3.0%; HR = 1.13 [0.77–1.65]; *p* = 0.546).

**Conclusions:**

In ACS patients treated with PCI, the cumulative rate of urgent revascularizations after 12 months is higher with ticagrelor compared to prasugrel, due to a significant increase in urgent revascularizations involving remote coronary vessels.

**Graphic abstract:**

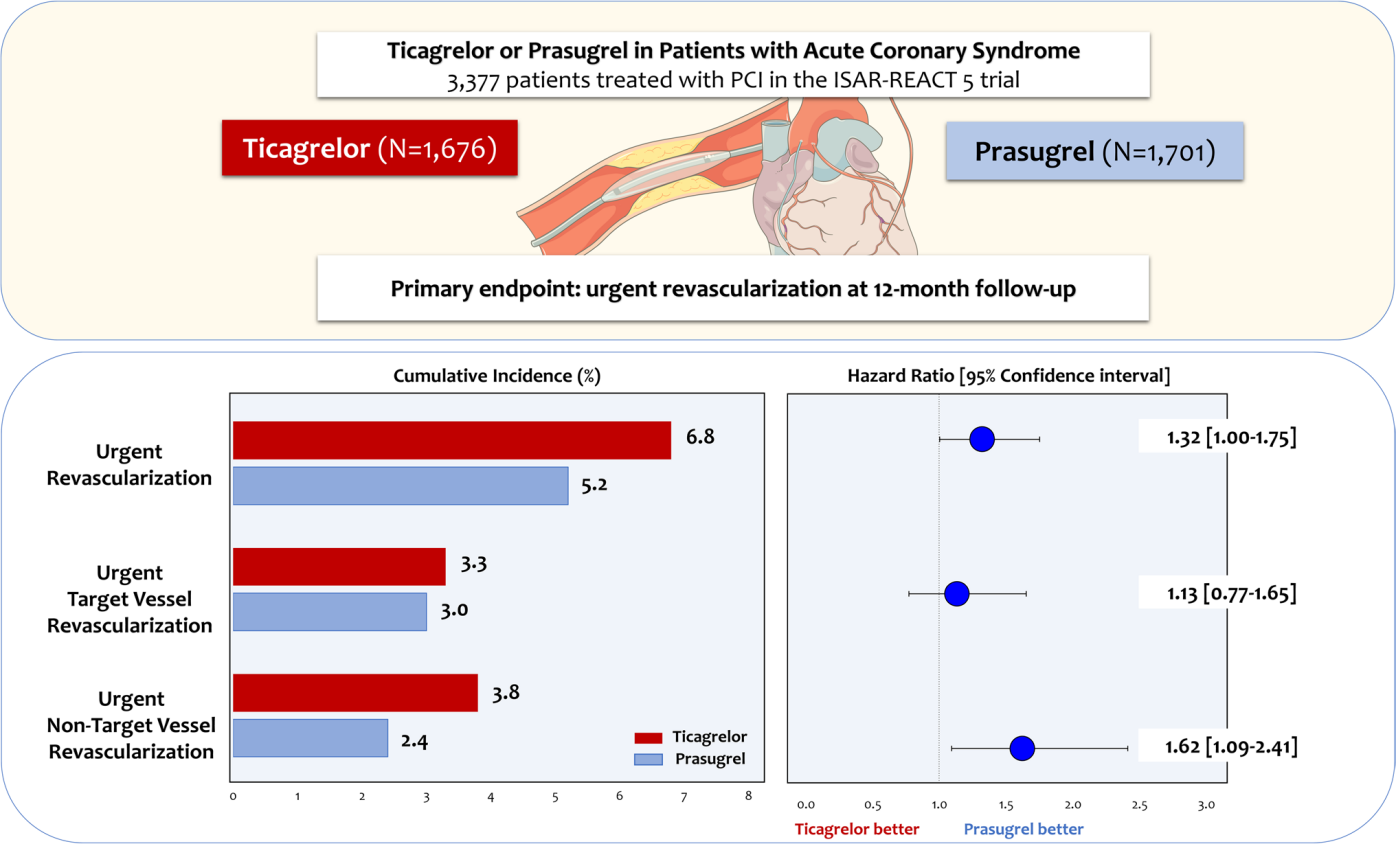

**Supplementary Information:**

The online version contains supplementary material available at 10.1007/s00392-024-02454-x.

## Introduction

The percutaneous coronary intervention (PCI) has shown great advancement both in terms of safety and efficacy in the contemporary era [1, 2]. Notwithstanding this, the number of readmissions for repeat revascularization following index PCI remains non-negligible [3, 4] and poses further risk of adverse clinical outcomes particularly in the presence of complex anatomy [5]. Previous studies reported that revascularizations of non-target vessels have a recognized role among subsequent procedures after index PCI [6, 7].

The potent P2Y_12_-inhibitors ticagrelor and prasugrel represent the antiplatelet therapies of choice in patients presenting with ACS without indication for lifelong oral anticoagulation [8]. The ISAR-REACT 5 randomized trial showed the superiority of prasugrel over ticagrelor in reducing the one-year incidence of ischemic outcomes without excess bleeding both in the overall trial population [9] and in the cohort receiving a PCI of a culprit lesion [10]. Interestingly, latest data lend support to different levels of off-target effects for ticagrelor and prasugrel involving endothelial function, inflammatory parameters, beyond platelet function inhibition [11, 12]. In this regard, whether ticagrelor and prasugrel affect the incidence of urgent revascularization involving both culprit and remote (non-treated) lesions remains unstudied. In fact, the whole spectrum of myocardial revascularizations has not been assessed in the 2 pivotal trials that compared prasugrel [13] or ticagrelor [14] with clopidogrel in patients with ACS. Against this background, the present analysis is the first to report data on the relative merits of ticagrelor versus prasugrel regarding urgent revascularization in patients with ACS managed invasively.

## Methods

### Study population and design

The current study is a post-hoc analysis of the ISAR-REACT 5 trial. Briefly, the ISAR-REACT 5 trial was an investigator-initiated, multicentre, randomized, open-label clinical trial. Patients with ACS (unstable angina, non-ST-segment elevation myocardial infarction – NSTEMI, or ST-segment elevation myocardial infarction – STEMI) planned to undergo invasive evaluation were eligible for enrolment [9, 15]. Patients were randomized only if they were eligible for both prasugrel and ticagrelor. Ethics committees from each participating institution approved the study protocol and the study complied with the Declaration of Helsinki and Good Clinical Practice. All patients provided written informed consent before enrolment.

### Treatment groups

Patients were randomly assigned to receive either ticagrelor or prasugrel following randomization. Patients in the ticagrelor group received a loading dose of 180 mg, followed by 90 mg twice daily. In the prasugrel group, patients received a loading dose of 60 mg, followed by 10 mg once daily. The loading dose of ticagrelor and prasugrel was administered as soon as possible after randomization and before coronary angiography in patients with STEMI. In patients who had ACS without ST-segment elevation, a prasugrel loading dose was administered once the coronary anatomy was known and before proceeding to PCI. Patients aged ≥ 75 years and those with a body weight < 60 kg received a maintenance dose of 5 mg prasugrel [16]. Finally, an initial single loading dose of 150–300 mg of intravenous or chewed aspirin was administered in both groups, followed by 75–100 mg once daily maintenance dose together with the study drug. A dual antiplatelet therapy with either ticagrelor or prasugrel in addition to aspirin was recommended for at least 12 months.

### Study endpoints, follow-up and monitoring

For the current study, the primary endpoint was the incidence of urgent revascularization (percutaneous or surgical). Urgent revascularization was defined as any unplanned hospital readmission and myocardial revascularization procedure due to symptoms or signs of acute ischemia. Secondary endpoint was the incidence of urgent revascularization procedures related to target vessel or non-target vessel. Target vessel revascularization (TVR) was defined according to the Academic Research Consortium (ARC)-2 Criteria as any repeat PCI or surgical bypass of any segment of the target vessel, including the target lesion [17]. Non-target vessel revascularization (NTVR) was defined as any revascularization of a remote vessel with respect to index PCI. Target lesion revascularization was defined as a repeat percutaneous intervention of the target lesion or bypass surgery of the target vessel performed for restenosis or other complication of the target lesion. The composite of death, MI, or stroke and Bleeding Academic Research Consortium (BARC) type 3 through 5 bleeding according to assigned therapy for the cohort of ACS patients who underwent PCI have previously been reported [10] and are shown in Supplemental Table 1. The primary and secondary endpoints of this post-hoc analysis were investigator-reported and were collected in the electronic case report forms, although they were not part of the primary analyses of the trial results. Urgent revascularization procedures associated with the occurrence of main study endpoints (death, MI, stroke and stent thrombosis – ST) were adjudicated by the Central Event Adjudication Committee.

Follow-up was scheduled at 30 ± 10 days, 6 ± 1 months and 12 ± 1 months. Patients were monitored either through telephone calls, structured follow-up letters, outpatient or hospital visits. In case of potential endpoint-related adverse events, we solicited source data from care practitioners in charge for the patient management.

### Statistical analysis

The current analysis is post-hoc since it was not pre-specified in the protocol of the parental trial. Continuous variables are expressed as mean ± standard deviation or median with interquartile range (IQR) and were compared between groups using Student’s t-test or the nonparametric Wilcoxon rank-sum test, respectively. Categorical variables are reported as frequencies and percentages and were compared using the χ2 or Fisher’s exact tests. Kaplan–Meier curves were created to estimate the event-free survival for each endpoint of interest. All endpoints were analyzed after accounting for the competing risk of death [18], and cumulative incidence functions were calculated by using the R-package *cmprsk* [19, 20]. Hazard ratios and 95% confidence intervals were calculated using Cox proportional hazards regression model. The model included the following factor variables as covariates: trial group, participating center, and clinical presentation. We performed a landmark analysis (using the 30-day time point as a landmark) to investigate a potential time dependence of the incidence of urgent revascularization according to assigned antiplatelet therapy. The analysis of the outcomes of interest for this study was mainly performed in the full analysis set according to the intention-to-treat principle. In addition, we assessed the treatment effect for primary outcome on the basis of what treatment the patients actually received, irrespective of the original randomization (on-treatment analysis). Hypothesis testing was performed at two tailed significance levels of 0.05. Statistical analysis was performed using the R 4.1.0 Statistical Package (The R Foundation for Statistical Computing, Vienna, Austria).

## Results

Of 4,018 patients enrolled in the ISAR-REACT 5 trial, 3,377 patients underwent PCI, 553 patients were treated conservatively, 83 patients underwent coronary artery bypass graft procedure and one patient in the ticagrelor group underwent surgery for aortic dissection. Treatment strategy was not available for 4 patients who withdrew consent. Of patients who underwent a CABG procedure after randomization (47 in the ticagrelor and 36 in the prasugrel group), only 5 patients (3 in the ticagrelor and 2 in the prasugrel group) underwent a revascularization procedure during the 1-year follow-up (*p* = 0.94).

### Baseline characteristics

In total, 3,377 of 4,018 patients (84.1%) were included in this analysis. Of them, 1,676 patients were assigned to ticagrelor and 1,701 to prasugrel. Baseline clinical characteristics in the overall cohort and in the treatment groups according to assigned therapy are displayed in Table 1. Of note, 523 out of 3,377 patients (15.5%) had prior MI and 749 (22.2%) had prior PCI, whilst 202 (6.0%) had prior surgical myocardial revascularization. The groups were well-balanced with regard to angiographic and procedural characteristics (Table 2). More than two thirds of participants had multivessel disease and more than half presented with a complex anatomy. Overall, the procedural success rate was 97.8%. Proportion of patients who discontinued treatment drugs and details of therapy at discharge are presented in Supplemental Table 2. The compliance with assigned antiplatelet therapy at different time points of follow-up is shown in Supplemental Fig. 1. The baseline and angiographic characteristics along with drug therapy at discharge for patients who were treated conservatively is shown in Supplemental Table 3.

**Table 1:** Baseline characteristics as per antiplatelet therapy

Characteristic	All (*N* = 3,377)	Ticagrelor (*N* = 1,676)	Prasugrel (*N* = 1,701)	*p* value
Age (years)	64.5 ± 12.0	64.4 ± 12.0	64.7 ± 12.0	0.522
Sex				0.976
*Women*	713 (21.1)	353 (21.1)	360 (21.2)	
*Men*	2,664 (78.9)	1,323 (78.9)	1,341 (78.8)	
Diabetes	743/3,375 (22.0)	376/1,675 (22.4)	367/1,700 (21.6)	0.575
*On insulin therapy*	240/3,375 (7.1)	120/1,675 (7.2)	120/1,700 (7.1)	0.958
Current smoker	1,189/3,363 (35.4)	584/1,669 (35.0)	605/1,694 (35.7)	0.690
Arterial hypertension	2,329/3,370 (69.1)	1,181/1,672 (70.6)	1,148/1,698 (67.6)	0.062
Hypercholesterolemia	1,946/3,371 (57.7)	978/1,672 (58.5)	968/1,699 (57.0)	0.391
Prior myocardial infarction	523/3,375 (15.5)	261/1,675 (15.6)	262/1,700 (15.4)	0.929
Prior percutaneous coronary intervention	749/3,374 (22.2)	374/1,675 (22.3)	375/1,699 (22.1)	0.890
Prior coronary artery bypass grafting	202/3,375 (6.0)	95/1,675 (5.7)	107/1,700 (6.3)	0.490
Cardiogenic shock	63 (1.9)	30 (1.8)	33 (1.9)	0.845
Systolic blood pressure (mmHg)	143 ± 25.0	143 ± 25.4	143 ± 24.6	0.329
Diastolic blood pressure (mmHg)	82.1 ± 14.3	82.2 ± 14.7	81.9 ± 13.9	0.539
Heart rate (beats/min)	76.4 ± 15.6	76.9 ± 15.6	75.9 ± 15.6	0.064
Body mass index (kg/m^2^)	27.8 ± 4.5	27.8 ± 4.6	27.8 ± 4.4	0.780
Body weight < 60 kg	151/3,351 (4.5)	81/1,668 (4.9)	70/1,683 (4.2)	0.374
Creatinine (µmol/L)	87.9 ± 28.6	87.6 ± 27.0	88.1 ± 30.1	0.612
Diagnosis at Admission				0.983
*Unstable angina*	277 (8.2)	136 (8.1)	141 (8.3)	
*Non-ST-segment elevation myocardial infarction*	1,532 (45.4)	761 (45.4)	771 (45.3)	
*ST-segment elevation myocardial infarction*	1,568 (46.4)	779 (46.5)	789 (46.4)	
Coronary angiography	3,377 (100.0)	1,676 (100.0)	1,701 (100.0)	> 0.999

Data are shown as mean ± standard deviation or counts (%)

Completeness of continuous data:

Systolic blood pressure was not available in 3 patients (1 in the ticagrelor group and 2 in the prasugrel group); diastolic blood pressure was not available in 16 patients (7 in the ticagrelor group and 9 in the prasugrel group); heart rate was not available in 2 patients (1 in each group); body-mass index was not available in 30 patients (11 in the ticagrelor group and 19 in the prasugrel group); creatinine level was not available in 6 patients (5 in the ticagrelor group and 1 in the prasugrel group). The remaining continuous data were complete

**Table 2:** Angiographic and procedural data as per antiplatelet therapy

Characteristic	All (*N* = 3,377)	Ticagrelor (*N* = 1,676)	Prasugrel (*N* = 1,701)	*p* value
Access site				0.702
*Femoral artery*	2,129 (63.0)	1,051 (62.7)	1,078 (63.4)	
*Radial artery*	1,231 (36.5)	618 (36.9)	613 (36.0)	
*Other*	17 (0.5)	7 (0.4)	10 (0.6)	
Number of diseased coronary arteries				0.676
*No obstructive CAD*	4 (0.1)	1 (0.1)	3 (0.2)	
*One-vessel disease*	1,100 (32.6)	555 (33.1)	545 (32.0)	
*Two-vessel disease*	1,008 (29.8)	491 (29.3)	517 (30.4)	
*Three-vessel disease*	1,265 (37.5)	629 (37.5)	636 (37.4)	
Multivessel disease	2,273 (67.3)	1,120 (66.8)	1,153 (67.8)	0.578
Left ventricular ejection fraction	51.2 ± 11.2	51.0 ± 11.3	51.4 ± 11.2	0.395
More than 1 lesion treated	1,173 (34.7)	569 (33.9)	604 (35.5)	0.360
Target vessel				0.598
*Left main coronary artery*	74 (2.2)	36 (2.2)	38 (2.2)	
*Left anterior descending coronary artery*	1,464 (43.4)	746 (44.5)	718 (42.2)	
*Left circumflex coronary artery*	691 (20.5)	346 (20.6)	345 (20.3)	
*Right coronary artery*	1,089 (32.2)	520 (31.0)	569 (33.5)	
*Bypass graft*	59 (1.8)	28 (1.7)	31 (1.8)	
Complex lesion (type B2/C)	1,987 (58.8)	979 (58.4)	1,008 (59.3)	0.642
TIMI before the intervention				0.281
*0*	1,176 (34.8)	592 (35.3)	584 (34.3)	
*1*	282 (8.4)	127 (7.6)	155 (9.1)	
*2*	747 (22.1)	361 (21.5)	386 (22.7)	
*3*	1,172 (34.7)	596 (35.6)	576 (33.9)	
TIMI after the intervention				0.465
*0*	33 (1.0)	17 (1.0)	16 (0.9)	
*1*	16 (0.5)	9 (0.5)	7 (0.4)	
*2*	87 (2.6)	50 (3.0)	37 (2.2)	
*3*	3,241 (96.0)	1,600 (95.5)	1,641 (96.5)	
Type of intervention				
*Drug-eluting stent*	3,040 (90.0)	1,497 (89.3)	1,543 (90.7)	0.197
*Bare-metal stent*	12 (0.4)	4 (0.2)	8 (0.5)	0.400
*Bioresorbable vascular scaffold*	195 (5.8)	99 (5.9)	96 (5.6)	0.799
*Drug-eluting balloon*	63 (1.9)	36 (2.2)	27 (1.6)	0.282
*Plain balloon angioplasty*	102 (3.0)	57 (3.4)	45 (2.7)	0.237
Maximal stent diameter (mm)	3.2 ± 0.5	3.2 ± 0.5	3.2 ± 0.5	0.497
Total stented length (mm)	30.5 ± 16.9	30.7 ± 16.8	30.3 ± 17.0	0.473
Successful percutaneous coronary intervention	3,302 (97.8)	1,640 (97.9)	1,662 (97.7)	0.866
Periprocedural antithrombotic medication				
*Aspirin*	3,035 (89.9)	1,503 (89.7)	1,532 (90.1)	0.752
*Unfractionated heparin*	3,177 (94.1)	1,581 (94.3)	1,596 (93.8)	0.584
*Low molecular weight heparin*	139 (4.1)	74 (4.4)	65 (3.8)	0.434
*Bivalirudin*	266 (7.9)	125 (7.5)	141 (8.3)	0.405
*Glycoprotein IIb/IIIa inhibitor*	417 (12.3)	219 (13.1)	198 (11.6)	0.227

Data are shown as mean ± standard deviation or counts (%)

Completeness of continuous data:

Left ventricular ejection fraction was not available in 198 patients (93 in the ticagrelor group and 105 in the prasugrel group)

*CAD* coronary artery disease, *TIMI* thrombolysis in myocardial infarction flow grade

### Clinical outcomes

The breakdown of numbers concerning urgent revascularization after index PCI as per assigned antiplatelet therapy is summarized in Table 3. The clinical outcomes for patients who were treated conservatively are shown in Supplemental Table 4. In this cohort, the risk of urgent revascularization (HR = 0.79 [0.30–2.06], *p* = 0.625) was not statistically different among treatment groups up to 12-month follow-up.

**Table 3:** Clinical outcomes

Outcome	Ticagrelor (*N* = 1,676)	Prasugrel (*N* = 1,701)	Hazard Ratio (95% CI)	*p* value
Urgent revascularization	113 (6.8)	88 (5.2)	1.32 [1.00–1.75]	0.051
Urgent target vessel revascularization	55 (3.3)	50 (3.0)	1.13 [0.77–1.65]	0.546
Urgent non-target vessel revascularization	63 (3.8)	40 (2.4)	1.62 [1.09–2.41]	0.017
Target lesion revascularization	69 (4.2)	66 (3.9)	1.08 [0.77–1.51]	0.673
Urgent target lesion revascularization	32 (1.9)	32 (1.9)	1.02 [0.62–1.66]	0.939

Data are number of events with cumulative incidence (%) after accounting for competing risk at 12-month follow-up

*CI* confidence interval

#### Urgent revascularization

**Fig. 1:**
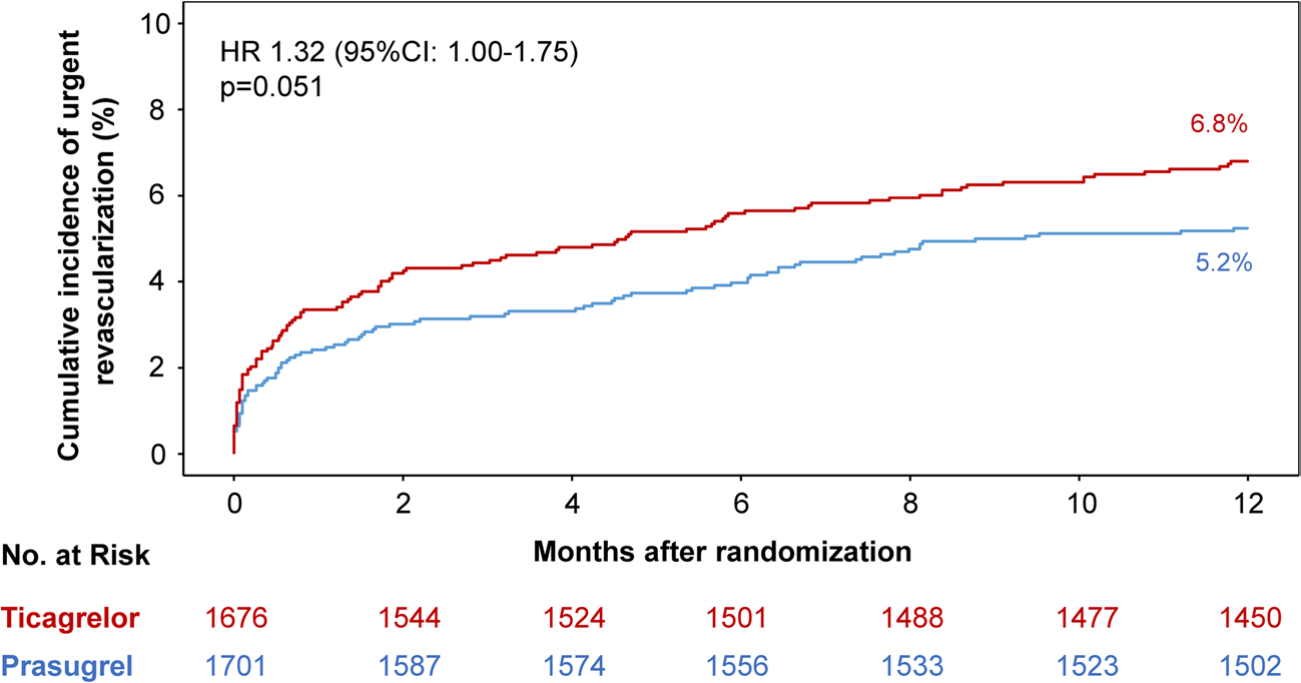
Twelve-month cumulative incidence of urgent revascularization after index PCI. CI = confidence interval; HR = hazard ratio

After 12 months, a total of 113 patients (6.8%) in the ticagrelor group and 88 patients (5.2%) in the prasugrel group underwent urgent revascularization (HR = 1.32 [1.00–1.75], *p* = 0.051; Fig. 1). In the on-treatment analysis, ticagrelor was associated with a significantly higher risk of urgent revascularization as compared to prasugrel (HR = 1.34 [1.01–1.78], *p* = 0.044; Supplemental Table 5). There were only 8 cases of myocardial infarction observed within 2 weeks after a revascularization procedure, 5 in the ticagrelor and 3 in the prasugrel group.

#### Urgent target vessel revascularization

**Fig. 2:**
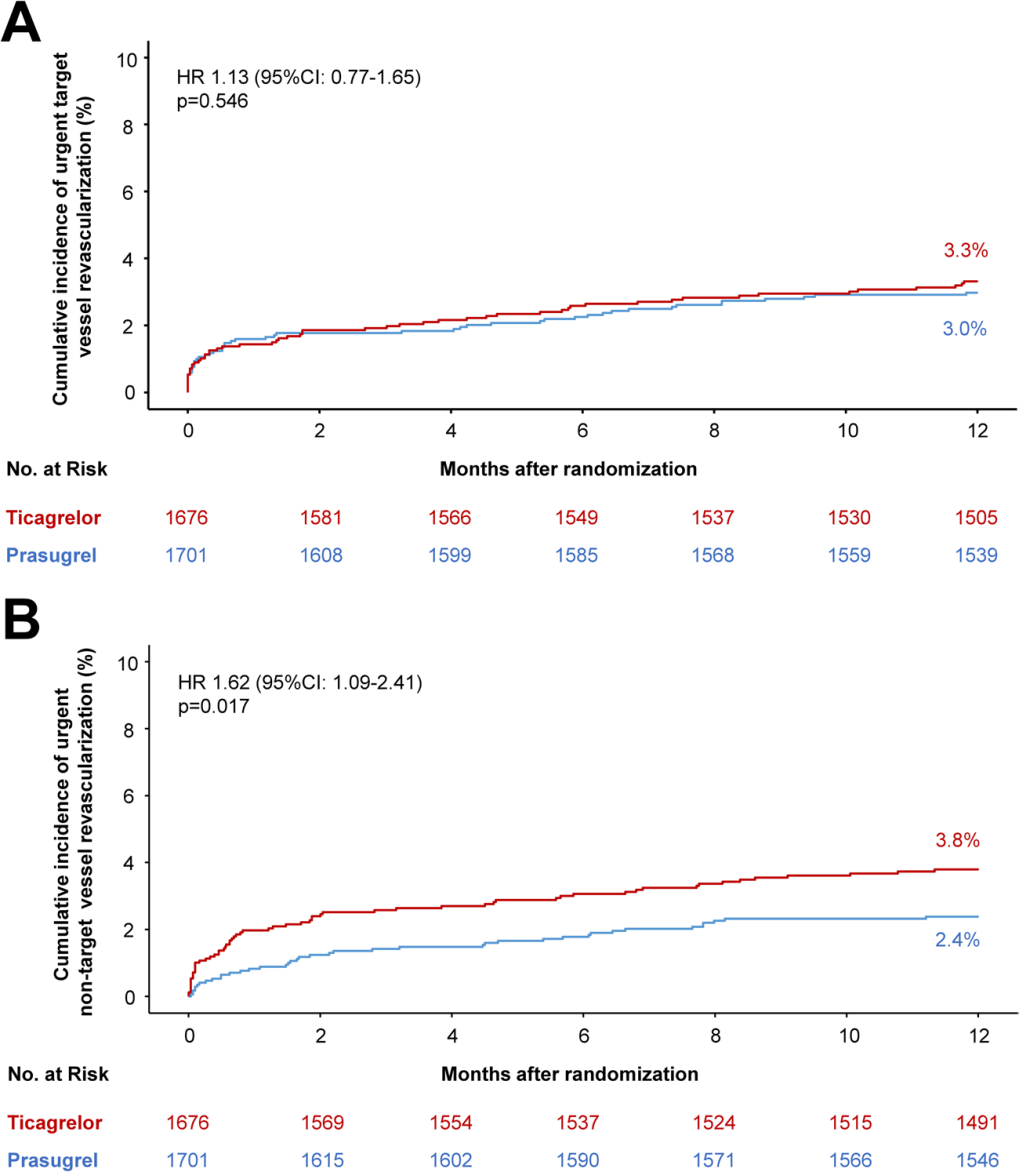
Twelve-month cumulative incidence of urgent (**A**) target- and (**B**) non-target vessel revascularization after index PCI. CI = confidence interval; HR = hazard ratio

After 12 months, 55 patients (3.3%) in the ticagrelor group and 50 patients (3.0%) in the prasugrel group underwent urgent TVR (HR = 1.13 [0.77–1.65], *p* = 0.546; Fig. 2A). In the on-treatment analysis, ticagrelor was associated with a comparable risk of urgent TVR as compared to prasugrel (HR = 1.08 [0.73–1.61], *p* = 0.694; Supplemental Table 5).

#### Urgent non-target vessel revascularization

After 12 months, urgent NTVR occurred in 63 patients (3.8%) in the ticagrelor group and 40 patients (2.4%) in the prasugrel group (HR = 1.62 [1.09–2.41], *p* = 0.017; Fig. 2B). The on-treatment analysis confirmed the higher risk for urgent NTVR associated with ticagrelor as compared with prasugrel (HR = 1.73 [1.16–2.59], *p* = 0.008; Supplemental Table 5).

#### Urgent target lesion revascularization

After 12 months, a total of 69 patients (4.2%) in the ticagrelor group and 66 patients (3.9%) in the prasugrel group underwent target lesion revascularization (HR = 1.08 [0.77–1.51], *p* = 0.673). Similarly, the incidence of urgent target lesion revascularization was comparable between the groups (1.9% vs. 1.9%; HR = 1.02 [0.62–1.66], *p* = 0.939).

### Landmark analysis

The result of the landmark analysis for the outcome urgent revascularization according to assigned antiplatelet therapies is displayed in Supplemental Fig. 2. From 0 to 30 days following PCI, 56 out of 1,676 patients (3.4%) in the ticagrelor group and 41 out of 1,701 patients (2.4%) in the prasugrel group underwent urgent revascularization (HR = 1.40 [0.94–2.10], *p* = 0.100). From 30 days to 12 months, urgent revascularization occurred in 57 patients (3.6%) in the ticagrelor group and 47 patients (2.9%) in the prasugrel group (HR = 1.25 [0.85–1.84], *p* = 0.256).

## Discussion

The key findings of this post-hoc analysis of the ISAR-REACT 5 trial are as follows:

i. Twelve months after index PCI, the incidence of urgent revascularization was higher in patients assigned to ticagrelor as compared to prasugrel.
ii. Approximately one half of urgent revascularizations were attributable to remote coronary vessels, with a significantly higher incidence of urgent NTVR procedures in patients receiving ticagrelor as compared to prasugrel.
iii. The risk of urgent TVR or TLR did not differ significantly between treatment groups.

Although current percutaneous technologies and potent antithrombotic therapies have an indisputable role in reducing adverse events in patients with unstable coronary artery disease (CAD) [1, 2, 21], this is the first study investigating the incidence and pattern of urgent revascularization in ACS patients treated with PCI and assigned to ticagrelor and prasugrel in a randomized trial. Importantly, the present analysis is important because it focuses on the clinically more relevant event of urgent revascularization, performed in both target and non-target vessel localisations. We showed that differences in the efficacy between ticagrelor and prasugrel might go beyond the composite endpoint of death, MI and stroke, the typical endpoint in trials of P2Y_12_-inhibitors. In our opinion, this study provides unique contribution to the assessment of optimal antiplatelet therapy in patients with ACS, and its results deserve careful discussion.

Firstly, despite improved secondary prevention measures in patients with established CAD, the progression of atherosclerosis in remote sites, unrelated to the culprit lesions, increase significantly the burden of revascularizations in patients with ACS. In keeping with this, our analysis shows a similar rate of urgent NTVR and urgent TVR events. The evidence concerning the proportion, the timing, and the type of adverse events referable to these remote lesions remain controversial [4, 6, 22, 23]. Zellweger et al. [23] reported a higher incidence of target vessel events compared to events attributable to remote vessels from 7 months up to 5 years in patients treated with earlier stent platforms. In a recent pooled analysis of 2 randomized trials, Coughlan et al. [6] reported that events related to non-culprit lesions make up for a higher proportion of total events at 10 years. At variance with our study, this latter analysis showed a higher incidence of target vessel related events up to one year post-PCI. The frequency of urgent NTVR observed in our study might possibly reflect a well-known overall limitation of coronary angiography. Patients with multivessel disease could have possibly presented with angiographically mild non-culprit lesions at the time of index PCI, whereas in reality, these were critical (or vulnerable) lesions with thin-cap fibroatheroma, a large plaque burden and/or stenosis prone to rupture and thrombosis [22, 24]. Furthermore, factors such as vessel geometry that might affect and influence the coronary disease development are not easily assessed through the two-dimensional planar projections of a coronary vessel [25]. A lower threshold to intracoronary imaging with in-vivo plaque-component characterization could be a valuable option to identify intermediate lesions amenable to revascularization independently from the degree of stenosis [26]. However, in the current practice, despite encouragement from consensus documents [27], the acutual penetration of intravascular imaging in clinical practice in Europe and the US remains relatively low [28, 29].

Secondly, it is worth to mention that the evidence of a high proportion of patients undergoing myocardial revascularization after a PCI for ACS has important clinical implications since earlier studies indicate a worse prognosis associated with repeat revascularizations [5, 30]. In fact, in the SYNTAX trial, including patients with complex coronary anatomy such as those with multivessel and/or left main disease, participants who underwent repeat revascularization had a significantly higher rate of adverse clinical events in comparison to those who did not [5]. Consistent with the results of the SYNTAX trial, in the current analysis over half of the patients who underwent myocardial revascularization among those included presented a complex coronary anatomy at the time of PCI of culprit lesions.

Finally in our study, the group of participants receiving ticagrelor had a higher number of urgent revascularization compared with the group receiving prasugrel. This correlates with a previous analysis from the ISAR-REACT 5 trial, in which a large proportion of MI events observed in the ticagrelor group were either type 1 (spontaneous), type 4a (PCI-related) or type 4b (ST-related) [9]. In addition, the higher incidence of urgent revascularization in the ticagrelor group was actually due to significantly more frequent urgent NTVR procedures. Of note, we observed a higher number of patients in the ticagrelor group who discontinued the study medication over the follow-up. This is in keeping with real-life data, in which the adherence to ticagrelor is diminished during maintenance phase [31], mostly because of extra-platelet inhibition side effects such as dyspnoea [32]. However, although interruptions of ticagrelor treatment may be harmful to patients because of the reversible mode of action of this antiplatelet agent, the on-treatment analysis performed for this study, including those patients actually taking the study medications, confirmed a higher risk for urgent revascularization with ticagrelor as compared to prasugrel.

### Study limitations

This is a post-hoc analysis of a randomized control trial and as such it suffers the common potential limitations associated with not pre-specified analyses. Accordingly, these results should be regarded as hypothesis generating. Second, information regarding intracoronary imaging or physiology at the time of revascularization was not routinely collected in the electronic case report forms in the primary trial. Thus, we have no data concerning intracoronary imaging or physiologies in the setting of both index and subsequent myocardial revascularization procedures. Third, this analysis did not account for recurrent revascularization events. Fourth, this report lacks detailed information concerning secondary prevention measures among patients with ACS, since this was not routinely collected among participants. In this regard, the association between secondary prevention medications (as those for lipid and glycemic control) and urgent revascularization in ACS patients treated with ticagrelor or prasugrel cannot be investigated. Finally, the ISAR REACT 5 trial was an open-label study, albeit with adjudication of clinical events carried out in a blinded manner.

## Conclusions

In patients with ACS treated with PCI, ticagrelor is associated with more urgent revascularizations after 12 months compared to prasugrel, mostly attributable to significantly more urgent revascularizations of remote coronary vessels.

### Supplementary Information

Below is the link to the electronic supplementary material.

Supplementary file1 (PDF 216 KB)

## Data Availability

The data underlying this article will be shared on reasonable request to the corresponding author.
