# Supplementary material for: Incidence and pattern of urgent revascularization in acute coronary syndromes treated with ticagrelor or prasugrel
Source: Clin Res Cardiol. 2024 May 13;113(7):1060–9. doi: 10.1007/s00392-024-02454-x (PMC11219404; doi:10.1007/s00392-024-02454-x)
Supplement: Supplementary file 1 — Supplementary file1 (PDF 216 KB) [file 392_2024_2454_MOESM1_ESM.pdf]

SUPPLEMENTAL APPENDIX

Incidence and pattern of urgent revascularization in acute coronary syndromes treated with ticagrelor or prasugrel

This online supplement contains the following items:

Supplemental Table 1. Clinical outcomes as per assigned antiplatelet therapy..................................2

Supplemental Table 2. Drug therapy at discharge and subsequent antithrombotic medications after discontinuation of assigned antiplatelet therapy. 3

Supplemental Table 3. Baseline, angiographic characteristics and drug therapy at discharge in patients treated conservatively as per assigned antiplatelet therapy. 4

Supplemental Table 4. Clinical outcomes in patients treated conservatively as per assigned antiplatelet therapy. 6

Supplemental Table 5. Clinical outcomes in patients as per assigned antiplatelet therapy using on-treatment analysis. 7

Supplemental Figure 1. Compliance with the assigned antiplatelet therapy at different time points of the follow-up. 8

Supplemental Figure 2. One-month landmark analysis of urgent revascularization following PCI. ..9

Supplemental Table 1. Clinical outcomes as per assigned antiplatelet therapy.

Characteristic	Ticagrelor (N=1,676)	Prasugrel (N=1,701)	Hazard Ratio (95% CI)	p value
**Composite of death, myocardial infarction, or stroke – no. (%)**	162 (9.8)	120 (7.1)	1.41 [1.11-1.80]	0.005
**Death from any cause**	79 (4.8)	64 (3.8)	1.28 [0.92-1.77]	0.147
Cardiovascular	54	52		
Noncardiovascular	25	12		
**Myocardial Infarction**	88 (5.3)	55 (3.3)	1.67 [1.19-2.34]	0.003
Type 1	45	31		
Type 2	3	3		
Type 4a	19	10		
Type 4b	20	11		
Type 5	1	0		
**ST-elevation myocardial infarction**	29	12		
**Stroke**	16 (1.0)	14 (0.8)	1.20 [0.58-2.46]	0.621
Ischemic	11	12		
Hemorrhagic	5	2		
**Definite or probable stent thrombosis**	25 (1.5)	20 (1.2)	1.28 [0.71-2.30]	0.413
**Definite stent thrombosis**	21 (1.3)	12 (0.7)	1.81 [0.89-3.68]	0.101
**BARC type 3 to 5 bleeding**	100 (6.0)	95 (5.6)	1.08 [0.82-1.43]	0.586
BARC 3a	52	48		
BARC 3b	33	37		
BARC 3c	6	2		
BARC 4	2	4		
BARC 5a	1	0		
BARC 5b	6	4		

Data are number of events with Kaplan-Meier estimates (%) or cumulative incidence (%) after accounting for competing risk at 12-month follow-up. BARC type 3 to 5 bleeding was evaluated in the intention-to-treat population. BARC=Bleeding Academic Research Consortium; CI=confidence interval.

Supplemental Table 2. Drug therapy at discharge and subsequent antithrombotic medications after discontinuation of assigned antiplatelet therapy.

**Characteristic***	**All (N=3,372)**	**Ticagrelor (N=1,673)**	**Prasugrel (N=1,699)**	**p value**
**Final diagnosis of acute coronary syndrome– no. (%)**	3,364 (99.8)	1,669 (99.8)	1,695 (99.8)	>0.999
Unstable angina	261/3,364 (7.8)	132/1,669 (7.9)	129/1,695 (7.6)	
NSTEMI	1,509/3,364 (44.9)	747/1,669 (44.8)	762/1,695 (45.0)	
STEMI	1,594/3,364 (47.4)	790/1,669 (47.3)	804/1,695 (47.4)	
**Therapy at discharge – no. (%) †**				
Aspirin	3,260/3,317 (98.3)	1,610/1,642 (98.1)	1,650/1,675 (98.5)	0.380
Ticagrelor	1,538/3,317 (46.4)	1,527/1,642 (93.0)	11/1,675 (0.7)	<0.001
Prasugrel	1,562/3,317 (47.1)	19/1,642 (1.2)	1,543/1,675 (92.1)	<0.001
Clopidogrel	183/3,317 (5.5)	75/1,642 (4.6)	108/1,675 (6.5)	0.022
Oral anticoagulant drugs	146/3,317 (4.4)	67/1,642 (4.1)	79/1,675 (4.7)	0.419
Beta blocking agents	2,875/3,317 (86.7)	1,430/1,642 (87.1)	1,445/1,675 (86.3)	0.520
ACE inhibitor/ARB	2,889/3,317 (87.1)	1,417/1,642 (86.3)	1,472/1,675 (87.9)	0.191
Statin	3,161/3,317 (95.3)	1,564/1,642 (95.2)	1,597/1,675 (95.3)	0.964
**Characteristic****	**All** (N=396)	**Ticagrelor** (N=218)	**Prasugrel** (N=178)	**p value**
**Therapy after discontinuation of study drug**				
Ticagrelor ††	13 (3.3)	0 (0.0)	13 (7.3)	<0.001
*Prasugrel ****	35 (8.8)	35 (16.1)	0 (0.0)	<0.001
Clopidogrel	216 (54.5)	115 (52.8)	101 (56.7)	0.489
Oral anticoagulation	78 (19.7)	38 (17.4)	40 (22.5)	0.259
None of the aforementioned medication	111 (28.0)	59 (27.1)	52 (29.2)	0.718

Data are shown as counts (%). * Not available for patients who withdrew consent before discharge. ** Percentages refer to patients who discontinued the study drugs during follow-up. † Shown for patients discharged alive, not available for patients who withdrew consent. †† Of 13 patients who switched from prasugrel to ticagrelor, 2 switched due to allergy, 8 due to decision of the attending physician, 1 due to Dyspnoea and 2 due to unspecific side effects to medication. *** Of 35 patients who switched from ticagrelor to prasugrel, 2 switched due to allergy, 2 due to bleeding, 1 due to Bradycardia, 8 due to decision of the attending physician, 17 due to Dyspnoea, 1 due to incompliance and 4 due to unspecific side effects to medication. ACE=angiotensin-converting enzyme; ARB=angiotensin receptor blocker; NSTEMI=non-ST-segment elevation myocardial infarction; STEMI=ST-segment elevation myocardial infarction.

Supplemental Table 3. Baseline, angiographic characteristics and drug therapy at discharge in patients treated conservatively as per assigned antiplatelet therapy.

	All (N=553)	Ticagrelor (N= 285)	Prasugrel (N= 268)	p value
**Baseline characteristics**				
**Age (years)**	64.5 ± 12.7	64.9 ± 12.8	64.2 ± 12.5	0.502
**Sex**				0.734
Women	230 (41.6)	121 (42.5)	109 (40.7)	
Men	323 (58.4)	164 (57.5)	159 (59.3)	
**Diabetes**	120 (21.7)	69 (24.2)	51 (19.0)	0.169
On insulin therapy	34 (6.2)	21 (7.4)	13 (4.9)	0.292
**Current smoker**	129/551 (23.4)	77/283 (27.2)	52 (19.4)	0.003
**Arterial hypertension**	419 (75.8)	213 (74.7)	206 (76.9)	0.628
**Hypercholesterolemia**	343/551 (62.3)	171/284 (60.2)	172/267 (64.4)	0.352
**Prior myocardial infarction**	95/552 (17.2)	41/284 (14.4)	54 (20.1)	0.096
**Prior percutaneous coronary intervention**	154 (27.8)	72 (25.3)	82 (30.6)	0.192
**Prior coronary artery bypass grafting**	41 (7.4)	19 (6.7)	22 (8.2)	0.597
**Cardiogenic shock**	2 (0.4)	1 (0.4)	1 (0.4)	1.000
**Systolic blood pressure (mmHg)**	144 ± 23.3	144 ± 23.5	144 ± 23.2	0.897
**Diastolic blood pressure (mmHg)**	81.1 ± 13.7	81.0 ± 14.5	81.1 ± 12.7	0.912
**Heart rate (beats/min)**	76.9 ± 16.8	77.3 ± 17.8	76.6 ± 15.7	0.631
**Body mass index (kg/m²)**	27.8 ± 4.8	27.6 ± 4.8	28.0 ± 4.8	0.351
**Body weight < 60 kg**	46/552 (8.3)	25/284 (8.8)	21 (7.8)	0.797
**Creatinine (µmol/L)**	88.2 ± 32.1	87.7 ± 30.7	88.7 ± 33.5	0.716
**Diagnosis at admission**				0.062
Unstable angina	209 (37.8)	99 (34.7)	110 (41.0)	
NSTEMI	268 (48.5)	138 (48.4)	130 (48.5)	
STEMI	76 (13.7)	48 (16.8)	28 (10.4)	
**Coronary angiography**	545 (98.6)	281 (98.6)	264 (98.5)	1.000
**Ticagrelor loading**	269 (48.6))	269 (94.4)	0 (0.0)	<0.001

**Prasugrel loading**	56 (10.1)	0 (0.0)	56 (20.9)	<0.001
**Angiographic characteristics**				
**Access site**				0.260
Femoral artery	332 (60.0)	164 (57.5)	168 (62.7)	
Radial artery	210 (38.0)	114 (40.0)	96 (35.8)	
Other	3 (0.5)	3 (1.1)	0 (0.0)	
No coronary angiography	8 (1.5)	4 (1.4)	4 (1.5)	
**Number of diseased coronary arteries**				0.220
No obstructive coronary artery disease	330/545 (60.6)	169/281 (60.1)	161/264 (61.0)	
One-vessel disease	76/545 (13.9)	44/281 (15.7)	32/264 (12.1)	
Two-vessel disease	59/545 (10.8)	24/281 (8.5)	35/264 (13.3)	
Three-vessel disease	80/545 (14.7)	44/281 (15.7)	36/264 (13.6)	
**Multivessel disease**	139/545 (25.5)	68/281 (24.2)	71/264 (26.9)	0.533
**Left ventricular ejection fraction**	55.3 ± 10.9	55.1 ± 11.1	55.6 ± 10.7	0.595
**Drug therapy at discharge***				
**Final diagnosis of acute coronary syndrome– no. (%)**				0.739
Unstable angina	81/196 (41.3)	46/114 (40.4)	35/82 (42.7)	
NSTEMI	97/196 (49.5)	56/114 (49.1)	41/82 (50.0)	
STEMI	18/196 (9.2)	12/114 (10.5)	6/82 (7.3)	
**Therapy at discharge – no. (%) †**				
Aspirin	405/552 (73.4)	211 (74.0)	194/267 (72.7)	0.788
Ticagrelor	59/552 (10.7)	58 (20.4)	1/267 (0.4)	<0.001
Prasugrel	45/552 (8.2)	0 (0.0)	45/267 (16.9)	<0.001
Clopidogrel	19/552 (3.4)	12 (4.2)	7/267 (2.6)	0.430
Oral anticoagulant drugs	34/552 (6.2)	15 (5.3)	19/267 (7.1)	0.467
Beta blocking agents	344/552 (62.3)	172 (60.4)	172/267 (64.4)	0.369
ACE inhibitor/ARB	403/552 (73.0)	207 (72.6)	196/267 (73.4)	0.913
Statin	402/552 (72.8)	201 (70.5)	201/267 (75.3)	0.246

Data are shown as mean ± standard deviation or counts (%). Completeness of continuous data: body-mass index was not available in 1 patient in the ticagrelor group; left ventricular ejection fraction was not available for 18 patients (10 in the ticagrelor group and 8 in the prasugrel group). The remaining continuous data were complete. * Not available for patients who withdrew consent or didn't survive the hospital stay before discharge. ACE=angiotensin-converting enzyme; ARB=angiotensin receptor blocker; NSTEMI=non-ST-segment elevation myocardial infarction; STEMI=ST-segment elevation myocardial infarction.

Supplemental Table 4. Clinical outcomes in patients treated conservatively as per assigned antiplatelet therapy.

Characteristic	Ticagrelor (N=285)	Prasugrel (N=268)	Hazard Ratio (95% CI)	p value
**Urgent revascularization**	8 (2.9)	9 (3.5)	0.79 [0.30-2.06]	0.625
**Composite of death, myocardial infarction, or stroke – no. (%)**	17 (6.1)	12 (4.6)	1.30 [0.63-2.81]	0.460
**Death from any cause**	8 (2.9)	6 (2.3)	1.25 [0.43-3.66]	0.682
**Myocardial Infarction**	7 (2.5)	5 (1.9)	1.31 [0.41-4.20]	0.650
**Stroke**	3 (1.1)	2 (0.8)	1.35 [0.23-8.09]	0.741
**BARC type 3 to 5 bleeding**	5 (1.8)	9 (3.4)	0.52 [0.17-1.55]	0.241

Data are number of events with Kaplan-Meier estimates (%) or cumulative incidence (%) after accounting for competing risk at 12-month follow-up. BARC type 3 to 5 bleeding was evaluated in the intention-to-treat population. BARC=Bleeding Academic Research Consortium; CI=confidence interval.

Supplemental Table 5. Clinical outcomes in patients as per assigned antiplatelet therapy using on-treatment analysis.

Characteristic	Ticagrelor (N=285)	Prasugrel (N=268)	Hazard Ratio (95% CI)	p value
**Urgent revascularization**	108 (6.8)	84 (5.2)	1.34 [1.01-1.78]	0.044
**Urgent target vessel revascularization**	50 (3.2)	48 (3.0)	1.08 [0.73-1.61]	0.694
**Urgent non-target vessel revascularization**	63 (4.0)	38 (2.4)	1.73 [1.16-2.59]	0.008

Data are number of events with cumulative incidence (%) after accounting for competing risk at 12-month follow-up.

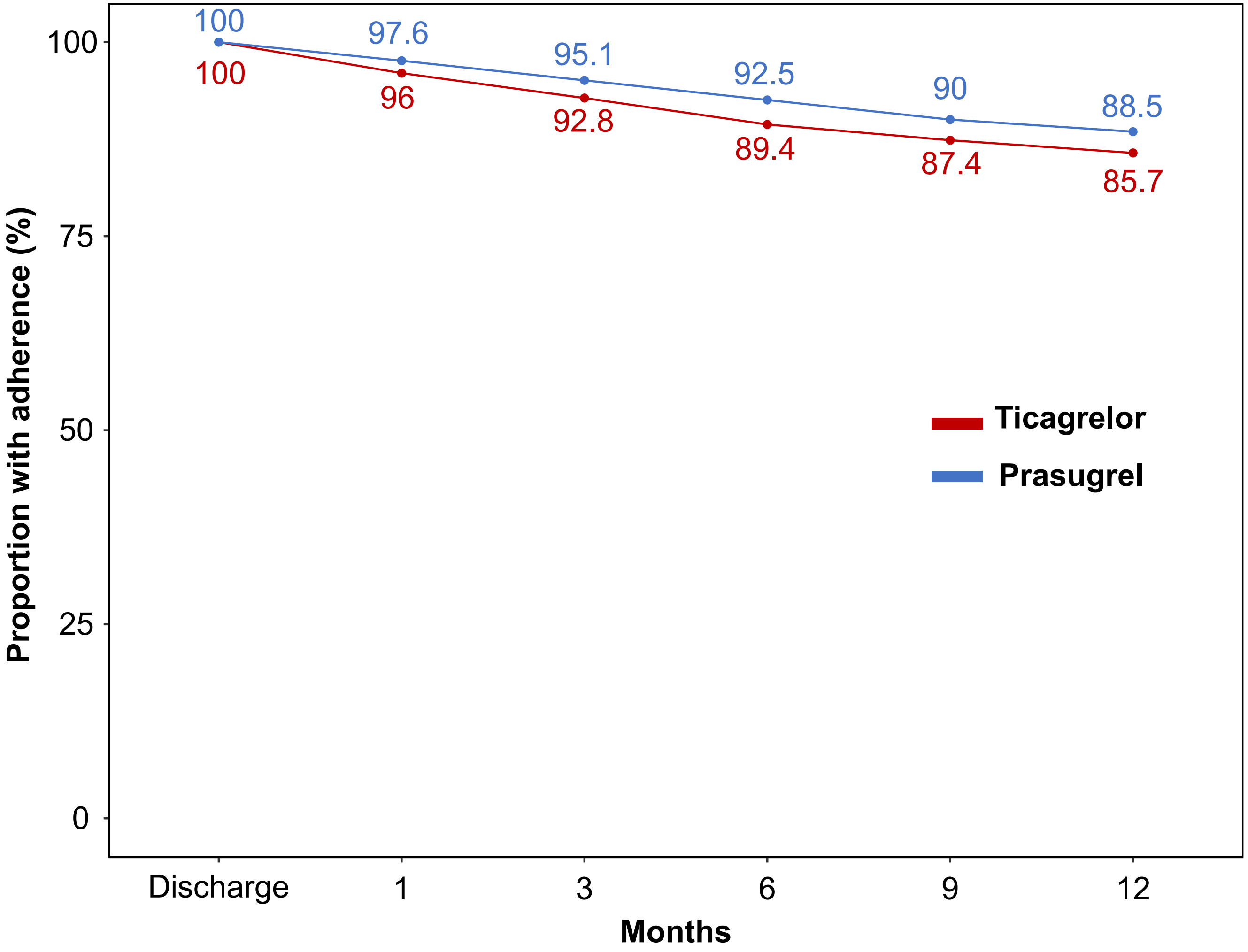

Supplemental Figure 1. Compliance with the assigned antiplatelet therapy at different time points of the follow-up.

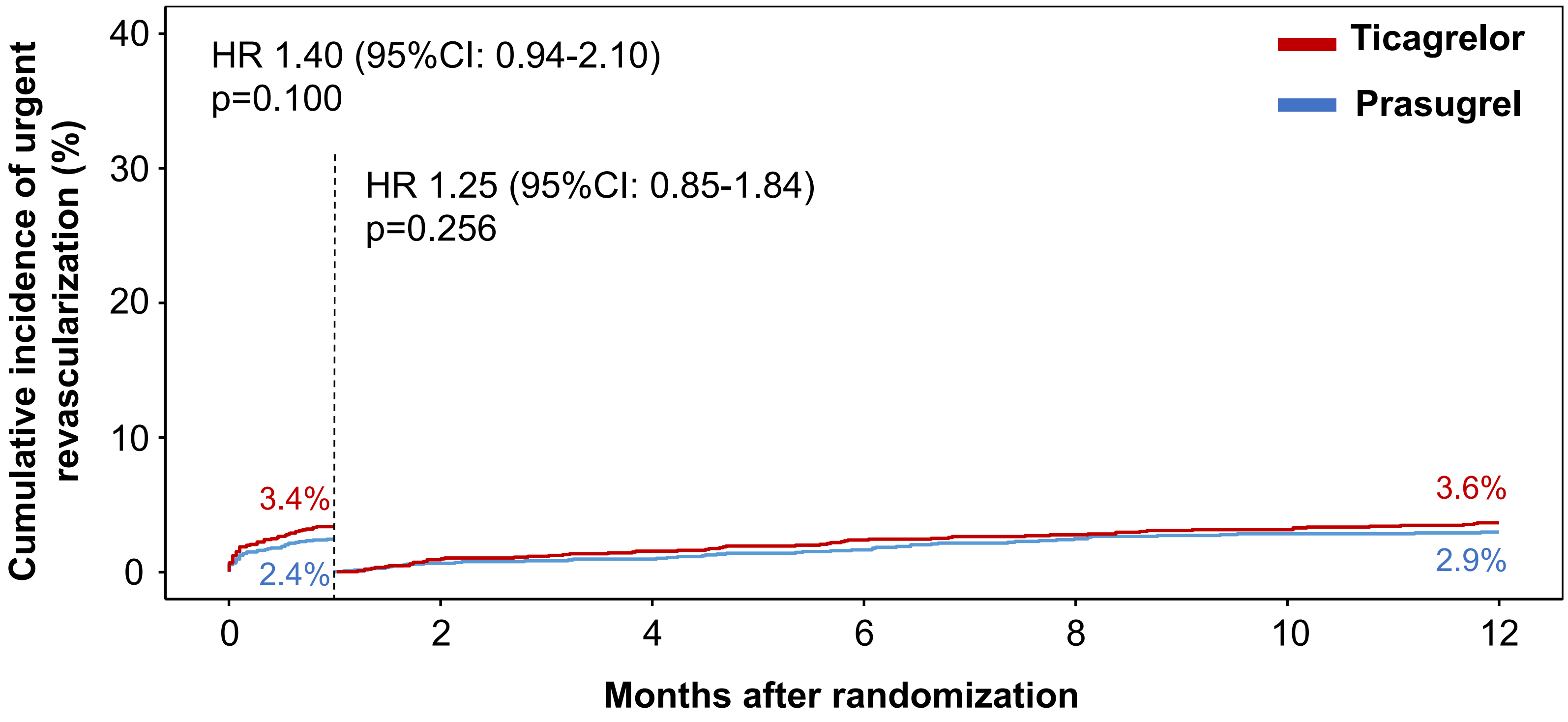

Supplemental Figure 2. One-month landmark analysis of urgent revascularization following PCI.

CI=confidence interval; HR=hazard ratio.
